# Placental CpG methylation of infants born extremely preterm predicts cognitive impairment later in life

**DOI:** 10.1371/journal.pone.0193271

**Published:** 2018-03-07

**Authors:** Sloane K. Tilley, Elizabeth M. Martin, Lisa Smeester, Robert M. Joseph, Karl C. K. Kuban, Tim C. Heeren, Olaf U. Dammann, T. Michael O’Shea, Rebecca C. Fry

**Affiliations:** 1 Department of Environmental Sciences and Engineering, Gillings School of Global Public Health, University of North Carolina, Chapel Hill, North Carolina, United States of America; 2 Department of Anatomy and Neurobiology, Boston University School of Medicine, Boston, Massachusetts, United States of America; 3 Department of Pediatrics, Boston Medical Center, Boston, Massachusetts, United States of America; 4 Department of Biostatistics, Boston University, Boston, Massachusetts, United States of America; 5 Department of Public Health and Community Medicine, Tufts University School of Medicine, Boston, Massachusetts, United States of America; 6 Department of Pediatrics, School of Medicine, University of North Carolina, Chapel Hill, North Carolina, United States of America; 7 Curriculum in Toxicology, School of Medicine, University of North Carolina, Chapel Hill, North Carolina, United States of America; Academic Medical Centre, University of Amsterdam, NETHERLANDS

## Abstract

**Background:**

The placenta is the central regulator of maternal and fetal interactions. Perturbations of placental structure and function have been associated with adverse neurodevelopmental outcomes later in life. Placental CpG methylation represents an epigenetic modification with the potential to impact placental function, fetal development and child health later in life.

**Study design:**

Genome-wide placental CpG methylation levels were compared between spontaneous versus indicated deliveries from extremely preterm births (EPTBs) (n = 84). The association between the identified differentially methylated CpG sites and neurocognitive outcome at ten years of age was then evaluated.

**Results:**

Spontaneous EPTB was associated with differential CpG methylation levels in 250 CpG sites (217 unique genes) with the majority displaying hypermethylation. The identified genes are known to play a role in neurodevelopment and are enriched for basic helix-loop-helix transcription factor binding sites. The placental CpG methylation levels for 17 of these sites predicted cognitive function at ten years of age.

**Conclusion:**

A hypermethylation signature is present in DNA from placentas in infants with spontaneous EPTB. CpG methylation levels of critical neurodevelopment genes in the placenta predicted later life cognitive function, supporting the developmental origins of health and disease hypothesis (DOHaD).

## Introduction

Preterm infants have an increased risk of cognitive impairment later in life [1]. In the Extremely Low for Gestational Age Newborns (ELGAN) cohort, indicators and antecedents of perinatal inflammation are associated with a range of neurodevelopmental outcomes in early and later childhood, including mental and motor impairment, behavioral problems, and cerebral palsy [2–6]. As the placenta mediates maternal-fetal interactions, it is likely that placental signaling is involved in these adverse developmental outcomes [7].

A potential mediator of gene and subsequent protein expression in the placenta is DNA methylation. DNA methylation of cytosine (CpG methylation) is an epigenetic modification that can influence gene expression levels without changing DNA sequences [7–10]. Placental CpG methylation has been reported to be a major mechanism by which the placenta dynamically responds to changing conditions throughout pregnancy and thus potentially affects long-term child health outcomes [7–11]. For example, CpG methylation in the placenta is associated with exposure to environmental factors including inorganic arsenic [12]. Further, placental CpG methylation is associated with newborn neurobehavior [13]. Interestingly, placentas derived from a male or female pregnancy have vastly different CpG methylation signatures [14], which may play a role in the observed differences in neurobehavioral outcome differences between males and females. Taken together, these data support that CpG methylation in the placenta is associated with in utero exposure to toxic substances and neurobehavior at birth.

The placenta serves as an environmental mediator as well as being the environment experienced by the gestating fetus. It plays a critical role in pregnancy, and abnormalities of the placenta have been linked to preterm delivery. Preterm birth is not considered to simply be the early induction of the same biological process as term birth [15]. In fact, two distinct subtypes of preterm birth have been identified and are suspected to have different underlying etiologies [16]. Indicated preterm birth is associated with placental dysfunction, including preeclampsia and intrauterine growth retardation. Spontaneous preterm birth, in contrast, is characterized by an inflammatory placental phenotype and is associated with pre-labor premature rupture of membranes, chorioamnionitis, and placental infections [16].

In the present study, the goal was to determine whether there are CpG methylation level differences in placental tissue from spontaneous versus indicated EPTB in the ELGAN cohort. As spontaneous EPTB is characterized by an inflammatory intrauterine environment, it was of interest to test whether genes that are differentially methylated in placentas from spontaneous EPTB also were associated with a neurodevelopmental outcome. The results indicate that methylation of genes in placental tissue predicts children’s cognitive function at ten years of age.

## Materials and methods

### Study subject recruitment

The recruitment process for the ELGAN study has been described in detail elsewhere [17]. Briefly, between 2002 and 2004, women who gave birth before 28 weeks gestational age at one of the 14 hospitals in five states in the United States were invited to participate in the study. A total of 1,249 mothers and 1,506 infants enrolled in the study. From these infants, 1,365 placentas were collected. Based on currently available placental epigenetic data and demographic information, a sub-cohort of 84 mother/infant pairs were investigated in the present study based on the child having intellectual deficit (n = 18), autism spectrum disorder without intellectual deficit (n = 18), or neither intellectual deficit or autism spectrum disorder (e.g. controls) (n = 48) [18, 19]. The lowest gestational age among infant participants for the current study was 23 weeks.

### Sample collection

Women were asked to contribute their placentas for the ELGAN study. After delivery, placentas were placed in a sterile exam basin and were biopsied under sterile conditions in a sampling room. To collect the chorion, the amnion was pulled back using sterile technique to expose the chorion at the midpoint of the longest distance between the cord insertion and the edge of the placental disk. A piece of tissue was removed by cutting at the base of the section after applying traction to the chorion and the underlying trophoblast tissue. The collected specimen was placed in a cryo-vial and immediately immersed in the liquid nitrogen. Placental samples were stored at -80°C until shipment to the University of North Carolina at Chapel Hill for processing as detailed [20].

### DNA extraction and Illumina 450K methylation assay

A subsection of placental tissue was cut from each frozen sample on dry ice, washed in sterile 1X PBS to remove residual blood. Subsections were then homogenized in Buffer RLT with β-mercaptoethanol (Qiagen, Valencia CA). The AllPrep DNA/RNA/miRNA Universal Kit (Qiagen, Valncia CA), in accordance with the manufacturer’s instructions, was used to isolate DNA and RNA sequences greater than 18 nucleotides in length. Isolated DNA was first bisulfite-converted using the EZ DNA methylation kit (Zymo Research, Irvine, CA). Converted DNA was then hybridized onto the Illumina HumanMethylation450 BeadChip (Illumina, Inc, San Diego, CA), which assesses the methylation levels of a total of 486,428 individual probes. Each probe measures the methylation level at a single CpG site. BeadChip microarray data were collected at Expression Analysis, Inc (Durham, NC; www.expressionanalysis.com). Methylation levels were calculated and expressed as *β* values (*β* = intensity of the methylated allele (M)) / (intensity of the unmethylated allele (U) + intensity of the methylated allele (M) + 100), as previously described elsewhere [21]. Batch effect was evaluated using principle component analysis (PCA) and was not a significant source of variation (p = 0.25). For data filtration, probes with high detection *P*-values (*P* > .01) were considered to be unreliable and removed from analysis (*n* = 24,591), as recommended by the manufacturer. Subsequently, data were normalized using the beta-mixture quantile (BMIQ) normalization methodology [22]. This was performed using the wateRmelon package (version 1.11.0) in R (version 3.2.3; The R Project for Statistical Computing) [23]. Probes that represent known single nucleotide polymorphisms (SNPs) (*n* = 84,124) and probes that are not associated with an annotated gene were removed, leaving a total of 286,410 probes for further analyses, representing 20,420 genes.

### Classification of pregnancy disorders

Disorders leading to EPTB may be classified into two groups, based on pregnancy complications, placental histology, and placental microbiology [16]. Spontaneous EPTB is characterized by preterm labor, prelabor premature rupture of membranes, placental abruption, or cervical insufficiency and is characterized by histologic chorioamnionitis and placental microbe recovery. Indicated EPTB is characterized preeclampsia or fetal indication/intrauterine growth restriction and is characterized by a paucity of organisms and inflammation but the presence of histologic features of abnormal placentation. The sample in the current study include n = 59 from spontaneous EPTB and n = 25 from indicated EPTB [16] selected for analysis based upon subjects with age 10 follow up and also currently available placental CpG methylation data.

### Cognitive assessment at ten years of age

When study participants were ten years of age, general cognitive ability (or IQ) was assessed with the School-Age Differential Ability Scales–II (DAS-II) Verbal and Nonverbal Reasoning scales [24]. Two subtests from the DAS-II and five subtests from the NEPSY-II were used to assess executive function [25]. DAS-II Recall of Digits Backward and Recall of Sequential Order measured verbal working memory. NEPSY-II Auditory Attention and Auditory Response Set evaluated auditory attention, set switching and inhibition. NEPSY-II Inhibition and Inhibition Switching assessed simple inhibition and inhibition in the context of set shifting, respectively. NEPSY-II Animal Sorting measured concept generation and mental flexibility. In order to obtain a unitary measure of cognitive function, a latent profile analysis (LPA) was used to identify subsets of study participants with similar profiles on all measures of IQ and executive functioning. Four profiles of cognitive function were identified: normal (34% of ELGAN cohort), low-normal (41%), moderately impaired (17%), and severely impaired (8%) [19, 26, 27].

### Statistical analysis of differential placental CpG methylation associated spontaneous versus indicated EPTB

To identify CpG sites that were differentially methylated between indicated EPTB (n = 25) and spontaneous EPTB (n = 59), mixed effect regression analysis was run for all annotated probes. Potential confounders were included in the model if they differed between placentas from indicated EPTB and spontaneous EPTB. To be most inclusive of potential confounders in the final regression models, p was set at < 0.20. Variables tested that did not differ between the two groups included maternal age, maternal race, maternal BMI, maternal education level, the number of participants on public health insurance, and maternal exposure to smoke during pregnancy (active or passive). Covariates included in the model were gestational age, infant sex, and infant birthweight. In order to control for multiple tests, FDR q-values were calculated. Significance was defined as an average beta difference ≥ |0.10|, which corresponds to approximately a 5% false positive rate, and a q-value < 0.05 [28]. As a secondary analysis the reference-free method of adjusting for cellular heterogeneity described in Houseman et al. 2014 was conducted using the RefFreeEWAS package in R [29]. This deconvolution method utilizes a surrogate variable analysis (SVA) that is data driven to identify latent variables as surrogates of cellular composition [29]. As in the prior analysis, the predictor variable tested was EPTB, and covariates included in the model were gestational age, infant sex and infant birth weight.

The distribution of differentially methylated probes (DMPs) was contrasted to the observed distribution of all annotated probes by intragene site: (i) from 200–1500 base pairs upstream of the gene transcription start site (TSS1500), (ii) within 200 base pairs upstream of the gene transcription start site (TSS200), (iii) in the 5’ untranslated region of the gene (5’UTR), (iv) in the first exon of the gene (1^st^ Exon), (v) in the body of the gene (Body), and (vi) in the 3’ untranslated region of the gene (3’UTR) [21]. Permutation testing was used to determine if the observed percentage of differentially methylated intragene sites significantly differed from the distribution of all annotated probes.

In order to examine the higher-level biological functions and processes, functional relationships among the differentially methylated genes were assessed using Ingenuity Network Analysis (IPA) (Ingenuity Systems^®^, Redwood City, CA, USA) and gene set enrichment analysis (GSEA). As the differentially methylated sites were tested in the context of approximately the entire genome (e.g. 286,410 probes representing 20,420/20,623 genes or 99% of the tested genome), the genome was selected as the background for the enrichment analyses. Canonical pathways enriched among this were analyzed and reported. Significance assessed using IPA was assessed using a right-tailed Fisher’s Exact test p-value < 0.0001. GSEA, which uses a rank-based analysis method to assess biological enrichment, was used as a second method to examine pathway enrichment [30]. GSEA examines discordant differences between two biological states by calculating an enrichment score within a ranked list. CpG methylation data along with relevant covariates have been deposited in the gene expression omnibus (GEO) database (GSE106089).

### Overrepresentation of transcription factor binding sites among DMPs

To examine whether the CpG sites identified as differentially methylated in spontaneous versus indicated EPTB had common transcriptional regulators, overrepresentation of transcription factor binding sites was performed using Genomatix (Genomatix Software Inc., Ann Arbor, MI). Overrepresentation of transcription factor binding sites was performed using the forward sequence corresponding to each of the probes identified as differentially methylated in spontaneous versus indicated EPTB. To determine whether a binding site is overrepresented, sequences for each probe compared to the MatBase of sequences. This is a database of transcription factor binding site motifs. The subsequent analysis identifies overrepresentation of individual transcription factor binding sites based upon a comparison to the genomic background using a Z-score calculation [31]. The most significant transcription factors are reported.

### Logistic regression of DMPs to later life cognitive function

Logistic regression analysis was performed in SAS (Cary, NC) to test whether the DMPs predicted later life neurocognitive function. Methylation levels were calculated as a proportion between 0 and 1. For the purposes of this analysis, β-values were adjusted to β-value*100 in order to examine the change in the odds ratio (OR) for each percent increase in methylation. This transformation does not change the underlying distribution of the data or the sensitivity of the model. The dependent variable was the binary outcome of either (i) no or low impairment (n = 39) or (ii) moderate or severe impairment (n = 43), derived from LPA, as described above. As confounders had been included in the first step of this analysis, the model was run with DNA methylation beta-values as the predictor variable. Sites of CpG methylation were considered to be significantly associated with LPA scores if the associated p-value was < 0.05. As the tests were run independently for the prediction of LPA score, a test for multiple test correction was not performed. Beta estimates, parameter-likelihood ORs and 95% confidence intervals (C.I.) for ORs are reported.

### Sensitivity analysis

In the ELGAN cohort, autism spectrum disorder was associated with intellectual deficit [18]. The subcohort used in the current study displayed a higher prevalence of autism spectrum disorder among study participants with intellectual deficit that is higher than would be typical of extremely preterm children with intellectual deficit. Thus, to assess whether this unusually high prevalence of autism spectrum disorder among children with intellectual deficit was a source of bias, the results were compared from regression analyses that either included or excluded the eighteen study participants with autism spectrum disorder.

## Results

### Study cohort

Maternal demographic data, pregnancy characteristics, and data on birth and later in life outcomes are presented for the ELGAN subjects used in this analysis (n = 59 spontaneous EPTB subjects, n = 25 indicated EPTB subjects) (Table 1) as well as for the larger ELGAN cohort (n = 1,506). Data are presented as the number (%) of subjects in the cohort unless otherwise noted. There were no significant differences in maternal age, maternal race, maternal body mass index (BMI), maternal education level, maternal status of public health insurance, Apgar and maternal exposure to smoke during pregnancy (active or passive) for spontaneous versus indicated EPTBs. The average gestational age of indicated EPTB infants was 1 week greater than that of spontaneous EPTB infants. However, the average birthweight of spontaneous EPTB infants was higher than that of indicated EPTB infants. Among the spontaneous EPTB group, there was also a higher proportion of male births (n = 44, 74.6%) than in the indicated EPTB group (n = 14, 56%).

**Table 1:** Subject characteristics. Maternal demographic data, pregnancy characteristics, and data on birth and later in life outcomes are presented for the ELGAN subjects used in this analysis. Data are presented as the number (%) of subjects in the cohort unless otherwise noted.

Characteristic	N = 25 Indicated EPTB Subjects	N = 59 Spontaneous EPTB Subjects	Student 2-sided t-test p-value (indicated vs. spontaneous EPTB)	N = 1506 Total ELGAN Subjects
	**Maternal Characteristics**			
**Maternal Age at Delivery (years)** Median (Range)	28.89 (17.0–40.5)	29.5 (16.0–39.4)	0.89	28.5 (13.2–47.3)
**Maternal Race** n (%)			0.48	
White	11 (44.0%)	27 (45.8%)		866 (57.4%)
African-American	9 (36.0%)	27 (45.8%)		427 (28.3%)
Other	5 (20.0%)	5 (8.5%)		187 (12.4%)
Unknown	0 (0.0%)	0 (0.0%)		26 (1.7%)
**Pre-pregnancy BMI (kg/m**^**2**^) Median (Range)	26.7 (15.2–43.2)	23.1 (18.6–72.1)	0.82	24.0 (13.2–72.1)
**Public Insurance** n (%)			0.31	
No	16 (64.0%)	30 (50.8%)		841 (55.7%)
Yes	9 (36.0%)	28 (49.2%)		594 (39.3%)
Unknown	0 (0.0%)	1 (1.7%)		71 (5.0%)
**Maternal Education** n (%)			0.71	
< = 12 years	5 (20.0%)	10 (19.9%)		242 (16.0%)
12–15 years	10 (40.0%)	33 (55.9%)		731 (48.4%)
16+ years	10 (40.0%)	14 (28.8%)		428 (28.3%)
Unknown	0 (0.0%)	2 (3.4%)		105 (9.9%)
**Smoking during Pregnancy** n (%)			0.21	
No	23 (92.0%)	47 (79.7%)		1212 (80.3%)
Yes	2 (8.0%)	10 (16.9%)		212 (14.0%)
Unknown	0 (0.0%)	2 (3.4%)		82 (5.7%)
	**Birth and Later Life Outcomes**			
**Extremely Preterm Birth (EPBT)** n (%)				
Yes	-	-		271(17.99%)
No	-	-		1235 (82.01^)
**Infant Sex** n (%)			0.12	
Male	14 (56.0%)	44 (74.6%)		799 (53.1%)
Female	11 (44.0%)	15 (25.4%)		707 (46.9%)
**Gestational Age (weeks)** Median (Range)	26.5 (23.5–27.5)	25.3 (23.6–27.6)	0.01	26.0 (23.0–27.6)
**Birth weight (g)** Median (Range)	640 (472–1025)	787 (544–1260)	1.28e-3	790 (99–1528)
**Cognitive Impairment** n (%)				
Normal	4 (16.0%)	12 (20.3%)		300 (34.3%)
Low-Normal	8 (32.0%)	15 (25.4%)		360 (41.1%)
Moderately Impaired	8 (32.0%)	14 (23.7%)		145 (16.6%)
Severely Impaired	5 (20.0%)	18 (30.5%)		69 (7.9%)
Unknown	0 (0.0%)	0 (0.0%)		632 (62%)
**Apgar 1 minute** Median (Range)	5 (1–8)	5 (1–9)	0.91	5 (0–9)
**Apgar 5 minute** Median (Range)	7 (3–9)	7 (2–9)	0.90	7 (0–10)

### Differences in placental DNA methylation of spontaneous versus indicated EPTB

**Fig 1:**
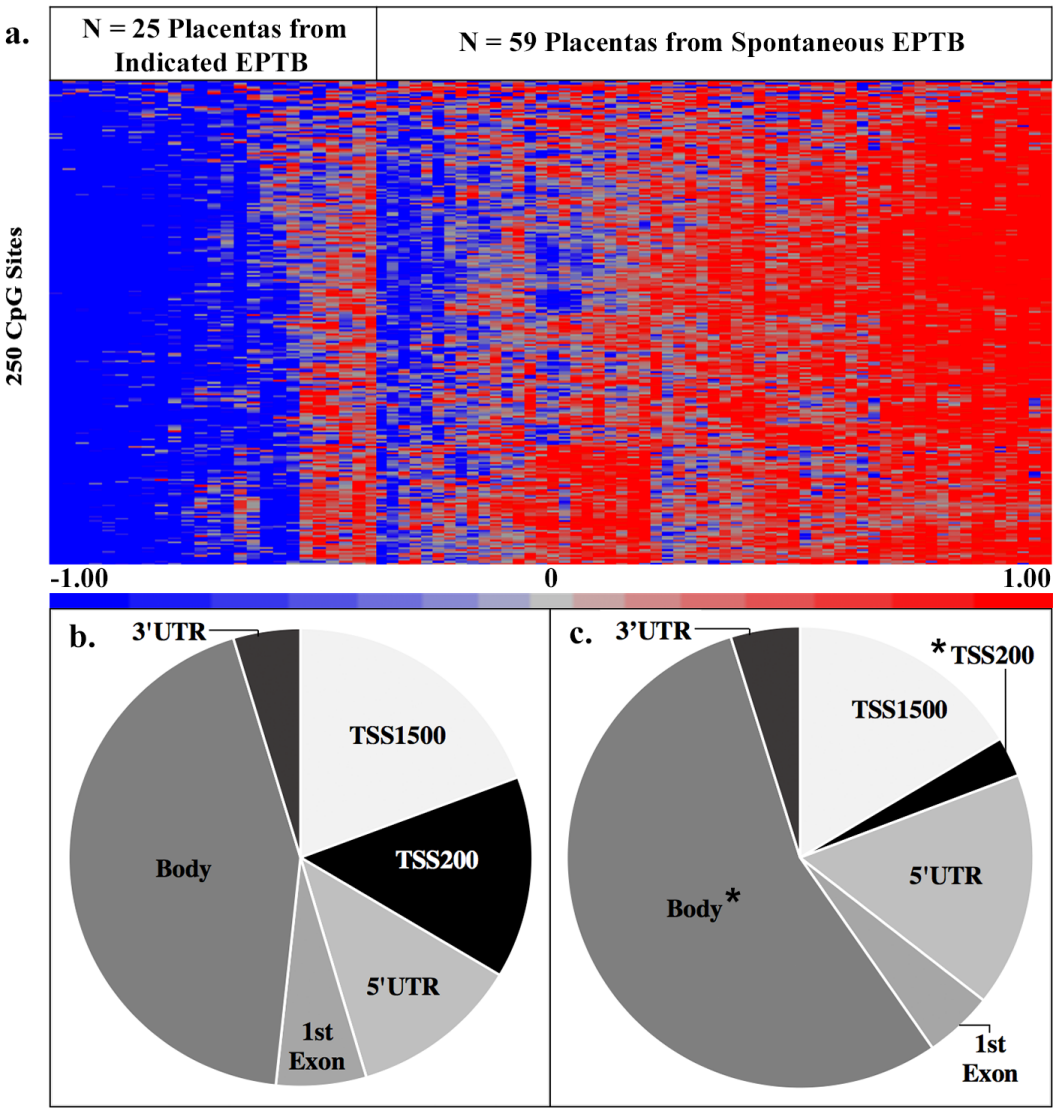
(a) Heatmap of 250 DMPs between placentas from indicated EPTB (n = 25) and spontaneous EPTB (n = 59). Beta-values were mean standardized and red indicates increased methylation levels, while blue indicates decreased methylation levels. Significance was defined as FDR q-value < 0.05 and an absolute beta difference ≥ |0.10|. (b) Intragene probe site distribution for all annotated probes (n = 286,410) contained on the Illumina HumanMethylation450 BeadChip. (c) Intragene probe site distribution for 250 DMPs between placentas from indicated EPTB (n = 25) and spontaneous EPTB (n = 59). The distribution of DMPs associated with intrauterine inflammation contained more probes located within the body region of genes and less probes located within the TSS200 region of genes than would be expected from a random sample of the total probe distribution, as indicated (*).

Mixed effect regression analysis was run where confounders were included in the mixed effect model analysis if they differed between subjects who had spontaneous versus indicated EPTB. Comparison of genome-wide CpG methylation levels in placentas from spontaneous versus indicated EPTB identified 250 differentially methylated probes (DMPs) (e.g. CpG sites) (S1 Table). These 250 DMPs were associated with 217 unique genes (S1 Table). Among these, 249 DMPs (99.6%) were hypermethylated, and one DMP (0.4%) was hypomethylated in association with spontaneous EPTB (Fig 1a). A set of three DMPs remained significant after SVA adjustment for cell type (S1 Table). As spontaneous EPTB is characterized by intrauterine inflammation [16], these hypermethylation marks could be marks associated with an inflammatory signature.

Interestingly, there were significantly more DMPs in the body region of genes and significantly less DMPs in the TSS200 region of genes than would be expected from the distribution of all annotated probes on the methylation array (Fig 1b and 1c). These results suggest that DNA methylation within the placenta is not random, and also that spontaneous EPTB is associated with hypermethylation outside of gene promoter regions.

### Hypermethylation in genes associated with neuronal development and function

Pathway-based (e.g. Ingenuity) analysis of the 217 unique genes revealed significant enrichment for several canonical pathways known to play a role in neuronal development (Table 2). Among the most significant were UDP-N-acetyl-D-galactosamine biosynthesis, an essential process in producing sialylated glycosphingolipids that are the primary sialic acid transporters in the central nervous system and plays an important role in mammalian development [32, 33]; CXCR4 signaling, in which CXCR4 and its ligand CXCL12 are both released from developing microglia to aid in axon guidance [34–36]; cholecystokinine/gastrin-mediated signaling, pathways implicated GABAergic neuronal function and in prenatal origins of autism-like neurodevelopmental disorders in rate, respectively [37, 38]; and protein kinase A signaling, which has been demonstrated to be necessary for maintenance of neuronal developmental plasticity [39] (Table 2). The enrichment of these pathways was validated using gene set enrichment analysis (GSEA).

**Table 2:** Enriched canonical pathways among the 217 DMP-associated genes.

Canonical Pathways Enriched Among N = 217 DMP-Associated Genes	p-value (IPA)	p-value (GSEA)	q-value (GSEA)	Associated Genes
UDP-N-acetyl-D-galactosamine Biosynthesis II	1.51E-04	5.36 E-5	4.75 E-3	*AGALE*, *HK2*, *HK1*
CXCR4 Signaling	2.63E-04	7.18 E-6	1.91 E-3	*PRKCE*, *GNAO1*, *ADCY7*, *GNA12*, *ITPR1*, *RHOF*, *PRKCZ*, *RAC1*
Cholecystokinin/Gastrin-mediated Signaling	5.37E-04	1.04 E-5	2.24 E-3	*PRKCE*, *EGFR*, *GNA12*, *ITPR1*, *RHOF*, *PRKCZ*
Protein Kinase A Signaling	5.89E-04	1.15 E-3	3.41 E-2	*FLNB*, *PRKCE*, *AKAP13*, *ADCY7*, *PTPRU*, *PDE7B*, *PTPN3*, *ITPR1*, *HIST1H1T*, *PRKCZ*, *ANAPC11*, *UBASH3B*
Endothelin-1 Signaling	6.03E-04	1.18 E-5	2.24 E-3	*PRKCE*, *PLA2G4E*, *GNAO1*, *ADCY7*, *GNA12*, *ECE1*, *ITPR1*, *PRKCZ*
Phospholipase C Signaling	6.61E-04	6.84 E-7	4.55 E-4	*PEBP1*, *PRKCE*, *PLA2G4E*, *ADCY7*, *ITPR1*, *RHOF*, *PRKCZ*, *RAC1*, *RALGDS*
Trehalose Degradation II (Trehalase)	9.77E-04	1.47 E-4	9.32 E-3	*HK2*, *HK1*

### Hypermethylated DMPs are enriched for binding sites for transcription factors associated with neuronal development

Enrichment analysis among the 250 DMPs revealed significant enrichment for binding sites of transcription factors with known roles in neurodevelopment. Interestingly, six of the eight most significant transcription factors were basic helix-loop-helix (bHLH) transcription factors (Table 3). Namely, these were aryl hydrocarbon receptor nuclear translocator (ARNT), basic helix-loop-helix domain containing, class B (BHLHB2), basic helix-loop-helix protein known as Dec1, Stra13, Sharp2 or BHLHE40 (DEC1), Hey-like bHLH-transcriptional repressor (HELT), v-myc avian myelocytomatosis viral oncogene neuroblastoma derived homolog (NMYC), and upstream transcription factor (USF). Specifically, BHLHB2, DEC1, and HELT Basic helix-loop-helix hairy and enhancer of split (Hes) family transcription factors, whose primary role is maintaining the population of self-renewing neural progenitor cells throughout neural development via the Notch signaling pathway [40, 41].

**Table 3:** Transcription factors with enriched binding sites among 250 DMPs. The most transcription factors with enriched binding sites among the 250 DMPs associated with spontaneous preterm deliveries due to intrauterine inflammation. The p-value was derived from a *Z*-score representing the overrepresentation of individual transcription factor binding sites and enrichment for individual transcription factor matrices compared against genomic background.

Transcription Factors with Overrepresentation of Binding Sites Among N = 250 DMPs	P-value	Associated Genes
BHLHB2*	8.34e-23	*CABLES1*, *CHD2*, *CHML*, *CTSC*, *ERN1*, *FLNB*, *HPCAL1*, *HSD3B1*, *KIAA1598*, *LAMC2*, *PLA2G4E*, *PLXNB1*, *PLXND1*, *RAC1*, *RDH13*, *SERINC2*, *SH3BP5*, *STX1A*, *SV2C*, *SYN2*, *TRIB3*, *VILL*
TCFAP2B	3.25e-22	*A2ML1*, *C1orf113*, *CMIP*, *EIF2C2*, *GNA12*, *HSD17B8*, *KRT19*, *MEGF11*, *MGAT5*, *MLLT1*, *OSBPL8*, *PLEKHA7*, *PRKCZ*, *PVT1*, *RDH13*, *SERINC2*, *SYNGR3*, *TGM1*, *TRIB3*, *WDFY2*
HELT*	3.05e-18	*BCL9L*, *CHD2*, *CTSC*, *ERN1*, *FLNB*, *HPCAL1*, *KIAA1598*, *PDE78*, *PLXNB1*, *PLXND1*, *PPARG*, *RAC1*, *SERINC2*, *SH3BP5*, *SV2C*, *SYN2*, *TMEM184A*, *VILL*, *ZP3*
HRE	1.17e-15	*CABLES1*, *DPRX*, *ERN1*, *FLNB*, *HEXB*, *HIC2*, *HPCAL1*, *KIAA1598*, *LAMC2*, *NEK6*, *OSBPL8*, *PLXNB1*, *PNPLA2*, *RAB20*, *RAC1*, *RDH13*, *RECQL5*, *SERINC2*, *SV2C*, *SYN2*, *VILL*, *ZSWIM4*
USF*	3.66e-15	*ASAP2*, *CHD2*, *CTSC*, *ERN1*, *FLNB*, *GJC2*, *HPCAL1*, *KIAA1598*, *MARCH14*, *PLA2G4E*, *PLXNB1*, *RAC1*, *SERINC2*, *SH3BP5*, *SV2C*, *SYN2*, *VILL*
DEC1*	3.99e-15	*BARXX2*, *CHD2*, *CHI3L2*, *ERN1*, *FLNB*, *HPCAL1*, *KIAA1598*, *PLA2G4E*, *PLXNB1*, *RAC1*, *SERINC2*, *SH3BP5*, *SV2C*, *SYN2*, *SYNGAP1*, *UNC119*, *VILL*
NMYC*	7.97E-15	*CHD2*, *ERN1*, *FLNB*, *HPCAL1*, *HSD3B1*, *KIAA1598*, *MARCH14*, *PLA2G4E*, *PLXNB1*, *RAC1*, *SERINC2*, *SH3BP5*, *STX1A*, *SV2C*, *SYN2*, *VILL*
ARNT*	7.97E-15	*C14orf181*, *CHD2*, *DPRX*, *ERN1*, *FLNB*, *HPCAL1*, *KIAA1598*, *NEK6*, *PLA2G4E*, *PLXNB1*, *PTPN3*, *RAC1*, *RDH13*, *SERINC2*, *SH3BP5*, *SV2C*, *SYN2*, *VILL*

* Indicates a bHLH transcription factor.

### Hypermethylation of DMPs predicts cognitive function at ten years of age

**Fig 2:**
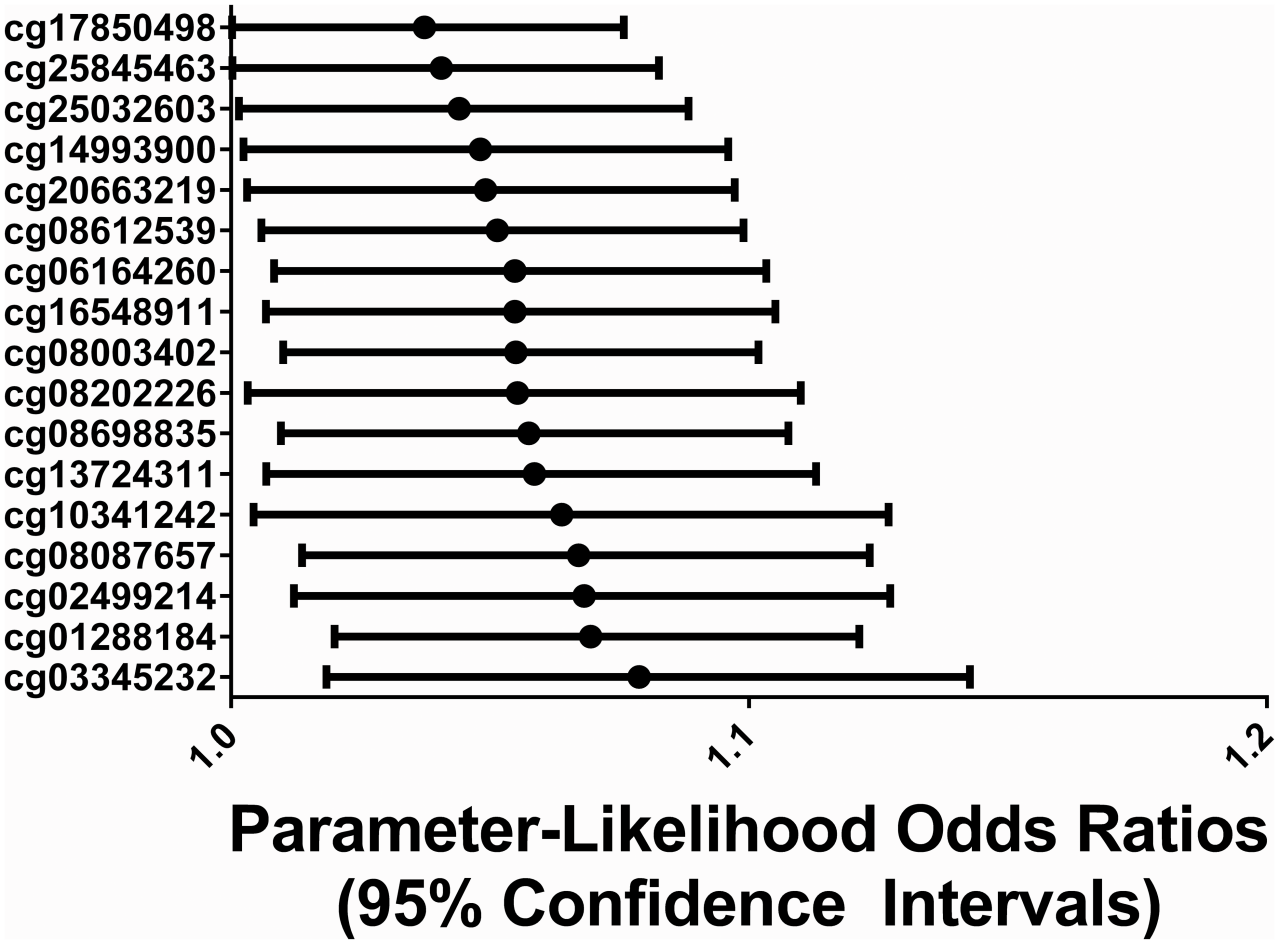
A total of 17 probe sites where increased placental CpG methylation predicted more severe cognitive impairment at ten years of age. These sites corresponded to 16 unique genes. The model represents the increase in the odds of moderate or severe cognitive impairment at age ten for every one percent increase in methylation at the probe site. Significance was defined as a p-value < 0.05 in a logistic regression model.

Logistic regression analysis revealed that 17 of the 250 DMPs predicted moderate to severe cognitive impairment at ten years of age (Fig 2, S2 Table). All of these sites were hypermethylated in association with spontaneous versus indicated EPTB. In addition, at all sites, higher levels of placental methylation predicted greater cognitive impairment at ten years of age (Fig 2). These 17 sites corresponded to 16 unique genes, many of which are involved in neuronal development and function. Among these were adenylate cyclase 7 (*ADCY7*), Cdk5 and Abl enzyme substrate 1 (*CABLES1*), G protein subunit alpha o1 (*GNAO1*), protein kinase C zeta (*PRKCZ*), retional dehydrogenase 13 (*RDH13*), ras homolog family member F, filopodia associated (*RHOF*), SH3 domain binding protein 5 (*SH3BP5*), and syntaxin 1A (*STX1A*). For each of these genes, a one percent increase in methylation at their respective probe sites predicted a 4–7% increase in the odds of moderate or severe cognitive impairment at age ten.

In a sensitivity analysis that excluded children with a diagnosis of autism spectrum disorder, 12 of the 17 sites (71%) found to be significant in the original model were confirmed to predict more severe neurocognitive impairment at ten years of age (S2 Table). It is likely that the other five sites failed to reach significance in the sensitivity analysis due to the lowered power of the analysis caused by the restricted sample size.

## Discussion

Infants born prematurely have a higher risk of later-life cognitive impairments than those born at term, and the severity of these adverse outcomes is inversely associated with gestational age [19]. Intrauterine inflammation and its antecedents are associated with both premature birth and preterm infants’ risk of later-life cognitive impairments [6]. Although the exact mechanism by which inflammation alters neurodevelopment is unknown, the current literature postulates that placental epigenetics may be an important determinant of changes in early-life neurodevelopmental programming in response to inflammation [7, 11]. For example, DNA methylation levels of the glucocorticoid receptor (NR3C1) and 11β-hydroxysteroid dehydrogenase type 2 (HSD11B1), have been associated with adverse newborn neurobehavior [8–10].

In the present study, a differential CpG methylation signature was observed between placentas from spontaneous EPTB versus indicated EPTB. Specifically, 250 DMPs representing 217 genes were identified in placentas from spontaneous EPTB. Interestingly, these genes are involved in biological pathways crucial to many aspects of neural development, including axon-glia interactions, neuron migration, and developmental neuronal plasticity [32, 36, 39]. Furthermore, CpG methylation levels of critical neurodevelopment-related genes in the placenta predicted later life cognitive function. Specifically, these were *ADCY7*, which is involved in the regulation of neural networks [42]; *GNAO1*, which is the predominant guanine nucleotide-binding protein found in the brain and mutations of which have been associated with severe developmental delays [43]; *PRKCZ*, which promotes axon differentiation and is involved in neuronal survival, differentiation, outgrowth, and synaptic plasticity [44, 45]; and *RHOF*, which is essential in the beginning of neuronal dendritic spine formation and also promotes neurite retraction [46]. The results from this study indicate that epigenetic changes in the placentas from spontaneous EPTB are associated with cognitive function later in life.

Prior evidence suggests that gene-specific patterns in differential DNA methylation likely result from altered transcription factor binding, known as the “transcription factor occupancy theory” [47, 48]. To examine the possibility that DMPs observed in spontaneous versus indicated EPTB resulted from the binding of common transcriptional regulators, the 250 DMPs were analyzed and an enrichment for basic helix-loop-helix (bHLH) transcription factors was identified. These transcription factors are known to play a critical role in neuronal development [41]. Previous research has reported an association between perinatal methylation of the bHLH transcription factor HES1 in the umbilical cord and childhood cognitive function and behavior at four years of age [49]. Furthermore, genes whose methylation levels were predictive of later cognitive impairment were predicted to be regulated by these transcription factors. For example, these genes included *CABLES1*, whose expression is necessary for neurite outgrowth and which is a substrate of c-Abl tyrosine kinase, an important kinase in neurulation [50, 51] and *STX1A*, a nervous-system specific protein that contributes to neurite outgrowth and has been associated with both autism and attention deficit/hyperactivity disorder [52, 53]. The data suggest that bHLH transcription factors may play a critical role in epigenetic programming in the placenta that affects later life cognitive development.

When interpreting the results of this study, several factors should be considered. First, the present study focuses on CpG methylation in the placenta and not brain tissue. It is established that there are tissue-specific patterns of CpG methylation [54] as well as CpG sites that display conserved methylation patterns across tissues [55]. The results of this study do not suggest that CpG methylation in the placenta would be similar to CpG methylation in the fetal brain. Instead, placental CpG methylation represents a type of “biological recording” of placental signaling pathways that are critical for fetal growth and development. This work contributes to a growing body of literature showing altered placental CpG methylation in relation to infant behavior and potential deficits in cognitive function [8, 9, 56–58]. Second, it is recently documented that there are significant sex-based differences in the placenta [14]. There are also established sex-based differences in neurodevelopmental outcomes in male and female adolescents [27]. To address the impact of sex as an influencing factor related to differential CpG methylation, sex was included as a covariate in the analysis. While underpowered to do so here, further research could further explore the relationship between placental DNA methylation and neurocognitive outcomes separately in male and female children. Lastly, while we have used *in silico* methods for placenta cell deconvolution as described previously [29], future research should aim to catalog DNA methylation patterns by placental cell type.

In summary, a CpG hypermethylation signature was identified in placental tissue from spontaneous EPTB that could mediate, at least in part, the relationship seen between preterm birth and adverse neurodevelopmental outcomes later in life. As survival rates of extremely premature infants have significantly improved with recent medical advances, the population of young children with an increased risk of cognitive impairment has grown and likely will continue to grow in the future [59]. These impairments represent a significant burden to public health, translating into billions of healthcare dollars, a lower quality of life of affected individuals and their families, and a reduction in societal productivity [59]. A total of 17 DMPs were identified between spontaneous and indicated EPTB that could be investigated for use as perinatal clinical epigenomic biomarkers of these later-life impairments. While current standard practice relies on childhood cognitive tests to identify cognitive impairment, identification of neonates at highest risk for adverse neurodevelopmental outcomes could present opportunities for earlier interventions to improve the outcomes of these children [60].

## Supporting information

S1 Table DMPs associated with EPTB. A total of 250 probes displayed differential methylation (DM) in spontaneous versus indicated EPTB. Gene and intragene region annotations are included. Significance was defined as FDR q-value < 0.05 and an absolute median beta difference ≥ |0.10|.
(XLSX)

S2 Table Logistic regression and sensitivity analysis. Logistic regression modeling identified 17 DMPs that predicted a significantly greater risk of neurocognitive impairment (LPA score = 3 or 4 versus LPA score = 1 or 2) at 10 year of age. In a sensitivity analysis excluding 18 autistic individuals, 12 (71%) DMPs remained significant.
(XLSX)
